# Reusable Photocatalytic Optical Fibers for Underground, Deep‐Sea, and Turbid Water Remediation

**DOI:** 10.1002/gch2.201700124

**Published:** 2018-02-21

**Authors:** Sara Teixeira, Bruno Magalhães, Pedro M. Martins, Klaus Kühn, Lluís Soler, Senentxu Lanceros‐Méndez, Gianaurelio Cuniberti

**Affiliations:** ^1^ Institute for Materials Science and Max Bergmann Center of Biomaterials TU Dresden 01062 Dresden Germany; ^2^ Centro/Departamento de Física da University of Minho Campus de Gualtar 4710‐057 Braga Portugal; ^3^ Centro de Engenharia Biológica University of Minho 4710‐057 Braga Portugal; ^4^ Institut de Tècniques Energètiques Departament d'Enginyeria Química and Barcelona Research Center for Multiscale Science and Engineering Universitat Politècnica de Catalunya EEBE 08019 Barcelona Spain; ^5^ BCMaterials Basque Center for Materials Applications and Nanostructures UPV/EHU Science Park 48940 Leioa Spain; ^6^ IKERBASQUE Basque Foundation for Science 48013 Bilbao Spain; ^7^ Dresden Center for Computational Materials Science (DCMS) TU Dresden 01062 Dresden Germany; ^8^ Center for Advancing Electronics Dresden (CFAED) TU Dresden 01062 Dresden Germany

**Keywords:** ciprofloxacin, hybrid materials, photocatalysis, titanium dioxide, wastewater treatment

## Abstract

An approach for underground, deep, and turbid water remediation is presented based on optical fibers with a photocatalytic coating. Thus, photocatalytic TiO_2_ P25 nanoparticles immobilized in a poly(vinylidene difluoride) (PVDF) matrix are coated on polymeric optical fibers (POFs) and the photocatalytic performance of the system is assessed under artificial sunlight. To the best of our knowledge, poly(methyl methacrylate)‐POF coated with TiO_2_/PVDF and the reusability of any type of POF for photocatalytic applications are not previously reported. The photocatalytic efficiency of the hybrid material in the degradation of ciprofloxacin (CIP) and its reusability are evaluated here. It is shown that 50 w/w% of TiO_2_ P25 achieves a degradation of 95% after 72 h under artificial sunlight and a reusability of three times leads to a loss of activity inferior to 11%. The efficient removal of ciprofloxacin and the stability of the POF coated with TiO_2_ P25 successfully demonstrate its suitability in the degradation of pollutants with potential application in regions with low light illumination, as in underground and deep water.

## 1 Introduction

Advanced oxidation processes (AOP) and adsorption have emerged as an effective alternative to conventional wastewater treatments to address environmental issues.^1^, ^2^, ^3^ In spite of the simplicity and cost effectiveness of adsorption, this method does not degrade the pollutant and may yield secondary pollution during disposal of adsorbent material.^4^, ^5^ Relatively to AOP, it involves the generation of hydroxyl radicals that are highly reactive and oxidize the pollutants.^6^, ^7^ This process includes treatments, such as ozonation, Fenton oxidation, and photocatalysis.^8^, ^9^ The latter is an inexpensive technique because it only requires a source of radiation (UV or sunlight) and a photocatalyst, which is typically a semiconductor material.^10^, ^11^ Among photocatalysts, titanium dioxide (TiO_2_) has shown to be effective in the removal of organic pollutants. Its remarkable photocatalytic activity and chemical/photostability are key properties to allow high efficiencies.^12^ Additionally, studies focused on the environmental toxicity of different catalysts have shown that TiO_2_ nanoparticles are less toxic than other catalysts,^13^ such as zinc oxide (ZnO) nanoparticles.^14^ In the last decades, many novel materials have been produced and investigated in the scope of photocatalysis. Most of these studies focus on overcoming the drawbacks of current photocatalysis limitations, such as the reduced spectral activation of TiO_2_ (only active under UV radiation) and the difficulty in recovering and reusing the nanocatalyst used in slurry systems. In the former case, the approaches are doping catalytic materials^15^, ^16^, ^17^, ^18^ or the production of nanocomposites^19^, ^20^ with reduced band gap and enhanced photoactivation under visible radiation. Concerning the reusability, the nanocatalysts are typically immobilized on substrates that prevent their loss and allow reusability. In this sense, the expensive and time‐consuming step for photocatalysts recovery becomes unnecessary.^21^, ^22^ Several substrates, such as glass,^23^, ^24^ stainless steel,^23^, ^24^ polymers,^25^, ^26^ zeolites,^27^, ^28^ silica^29^, ^30^ and magnetic particles,^31^ among others, have been used in the immobilization of photocatalytic materials.

Despite the significant and extensive research on the issues above mentioned, other relevant challenges still need to be addressed to apply photocatalysis to unexplored and highly important environments. For instance, the turbidity of the polluted water is one of the most important factors to consider in a photodependent reaction as photocatalysis.^32^, ^33^ The presence of particles in suspension (e.g., solids, inorganic and fecal material) can scatter the light or completely impede its penetration, leading to a reduction of the efficiency of the system.^34^, ^35^ Further, these suspended particles can act as active sites for the adsorption of microorganisms and contaminants, disabling the light‐driven disinfection and pollutants degradation.^36^, ^37^ Additionally, in the photocatalytic slurry systems, increasing the concentration of the photocatalytic nanoparticles—to increase the photocatalytic efficiency—can cause undesirable turbidity that just like the pollutant particles will reduce the light penetration and thus the photocatalytic efficiency.^38^, ^39^ The removal of turbidity is a costly and time‐consuming pretreatment, such as coagulation‐flocculation^40^, ^41^ and membrane filtration.^37^, ^42^ Moreover, the coagulants (iron and aluminum salts) used to change the polluted water pH can be ecotoxic and generate large sludge volumes.^43^, ^44^, ^45^ Concerning the filtration processes, membrane fouling is one of the major drawbacks.^46^, ^47^

Other challenges are two emerging environmental problems: the remediation of contaminated groundwater and deep‐sea water (> 200 m). In the past, deep‐sea water was considered an uncontaminated environment.^48^ However, the intense research in this area indicates that both environments are affected by the increasing anthropogenic actions like agriculture, landfills, irrigation water, oil drilling, sea mining, and trawling.^49^, ^50^ Recent studies indicate that deep‐sea can accumulate contaminants like oil^51^, ^52^ and other persistent organic pollutants.^48^ Similarly, studies about groundwater pollution have revealed contamination with polycyclic aromatic hydrocarbons,^53^ fuel hydrocarbons, chlorinated ethylenes, and nonchlorinated solvents.^54^ The existing technologies to remediate groundwater (e.g., pump and treat, oxidation, biodegradation, and adsorption) are expensive and inefficient.^55^, ^56^, ^57^ This lack of efficient technologies to remediate remote polluted water increases the demand for new materials and efficient technologies.

As a possible solution, the use of optical fibers as a substrate for photocatalyst immobilization can overcome the mentioned limitations, enabling the utilization of the photocatalytic process in turbid, ground, or deep‐sea polluted water. In this system, the optical fibers play a double role: (1) as photocatalyst support and (2) as an efficient light transmission tool. This approach avoids light loss caused by turbidity because the light directly reaches the photocatalyst immobilized on the surface of the optical fibers and since the photocatalyst is immobilized, it is possible to reuse the material, reducing the overall process costs.^46^ Marinangeli and Ollis^58^, ^59^, ^60^ first proposed the use of quartz optical fibers with TiO_2_ deposition for remote light transmission and as solid support for photocatalysts in the late 1970s. Later, Hofstadler et al.^61^ designed a TiO_2_‐coated quartz fiber reactor and used it in the degradation of 4‐chlorophenol in water. More recently, polymeric optical fibers (POFs) of poly(methyl methacrylate) (PMMA) have been investigated, mainly due to their high mechanic flexibility, thermal stability, visible light transmission, simple handling, and low cost.^6^, ^62^, ^63^, ^64^, ^65^ The practicability of the POF should be highlighted since quartz optical fibers are more expensive and fragile. The first photocatalytic application of PMMA optical fibers with immobilized TiO_2_ nanoparticles on the surface was reported by Joo et al.^6^ Despite the promising results, the weak immobilization of the catalyst onto the POF surface did not allow efficient reuse of the material and leads to secondary pollution due to nanoparticle detachment from the POF surface.

The lack of reusable photocatalytic materials demands for new and robust coatings to efficiently immobilize the catalyst on the surface of the POF. In this sense, polymers as embedding matrices arise as an interesting approach, as they are chemically and mechanically stable, inexpensive, exhibit high durability, and may display a porous microstructure, suitable for photocatalytic applications.^66^, ^67^ These features make polymers one of the most used coating materials.^68^, ^69^, ^70^

In this scope, the present paper reports on the first study on a reusable photocatalytic polymeric coating in POF employed for degrading organic pollutants. Poly(vinylidene difluoride) (PVDF) was used to immobilize TiO_2_ P25 nanoparticles onto the surface of the PMMA‐POF due to its high resistance and the possibility to tailor the degree of porosity and pore size.^71^ Ciprofloxacin (CIP), a fluoroquinolone antibiotic, was used as a model pollutant to assess the photocatalytic efficiency and reusability of the produced materials.

## 2 Results and Discussion

### 2.1 Characterization of the Coating

**Figure 1:**
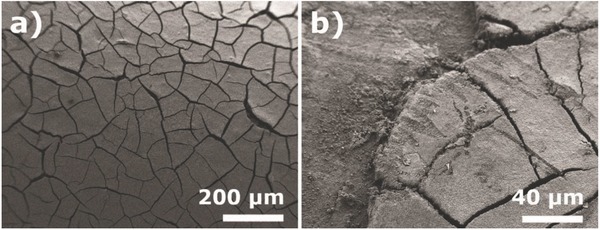
SEM images of the surface of an aqueous TiO_2_ solution coated on POF, 100× (a) and 500× (b).

To understand and improve previously reported approaches, scanning electron microscopy (SEM) images (**Figure** **1**) show a POF surface coated with TiO_2_ dispersed in water in which cracks (Figure 1a) and coating detachment (Figure 1b) were observed, and therefore, a smooth and stable coating layer was not obtained. The TiO_2_ coating was delaminated when introduced in water caused by the water flow. Agglomeration of the TiO_2_ particles and delamination of the coating were previously reported by Peil and Hoffman.^72^ It is to notice that the detachment of the photocatalyst decreases the POF photocatalytic efficiency limiting its reusability. Further, nanoparticles detachment is also the cause of undesirable secondary pollution.

Given the problems related to the aqueous TiO_2_ coating, several TiO_2_/PVDF coating solutions were then prepared. The optimization process of this coating addressed three parameters: the concentration of the PVDF and the TiO_2_ nanoparticles as well as the thickness of the coating. First, it was tested the optimum PMMA‐POF coating to posterior immobilize the TiO_2_ nanoparticles. Therefore, different polymer concentrations (3, 5, 10, and 15 w/w%) were tested. Only the 15% PVDF sample yielded a homogeneous and complete coating of the POF tip immersed within the PVDF solution. Hence, this polymer concentration was used to immobilize the TiO_2_ nanoparticles on the POF.

The effect of the concentration of TiO_2_ nanoparticles on the morphology and adhesion of the coating was assessed with 25, 40, and 50 w/w% TiO_2_ in 15 w/w% of PVDF by the tape test. The coatings with the concentrations of 40 and 50 w/w% of TiO_2_ exhibited the lowest coating weight losses, on the other hand, the coatings with a concentration of 25 w/w% of TiO_2_ yielded the highest mass loss, 0.7 mg. Thus, the loadings of 50 w/w% of TiO_2_ in 15 w/w% of PVDF were selected due to the highest concentration of catalyst and the higher stability of the coating (Figure S1, Supporting Information).

**Figure 2:**
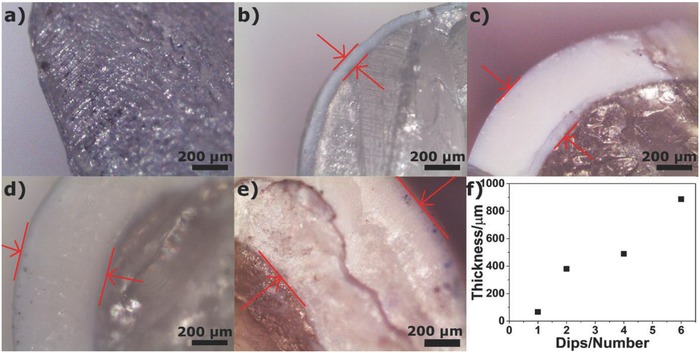
Microscope images (amplification of 50×) of a commercial PMMA optical fiber (a); coated with 50% w/w TiO_2_/PVDF by one dip (b), two dips (c), four dips (d), and six dips (e). The average thickness of the coating versus the number of dips is represented in (f).

To investigate the coating thickness one, two, four, and six consecutive dips were performed and the cross‐sections of the coated POF were inspected with an optical microscope (**Figure** **2**).

The coating thickness increases with the number of dips (Figure 2). Figure 2f summarizes the average coating thickness, as 66 ± 13, 385 ± 5, 489 ± 3, and 887 ± 8 µm were measured for one, two, four, and six dips, respectively (Figure 2b–e). The increase of the coating thickness by increasing the number of dips was previously reported.^73^, ^74^, ^75^, ^76^ The larger thickness found in the multicoating tests are related to the withdraw velocity of the POF and the viscosity of the solutions.^74^, ^75^ The thickness of the coating is critical for photocatalytic applications, as thicker coatings represent a drawback because of the higher distance between the inner layers—where the visible radiation intensity is higher as it is closer to the PMMA core that transports the radiation—and the outer layers, in contact with the pollutant.^76^ Therefore, the ideal thickness should provide a robust and lasting coating and allow the photocatalytic activation all along the photocatalytic coating. Taking this into account, two dips were used for the photocatalytic tests as it assures the best tradeoff between the thickness and light activation of the coating layers. The coatings obtained from four and six dips are too thick and the radiation is unable to cross all the coating. In this situation, the external layer is not activated and the hydrophilic behavior of the coating and consequently the photocatalytic process will be compromised.

**Figure 3:**
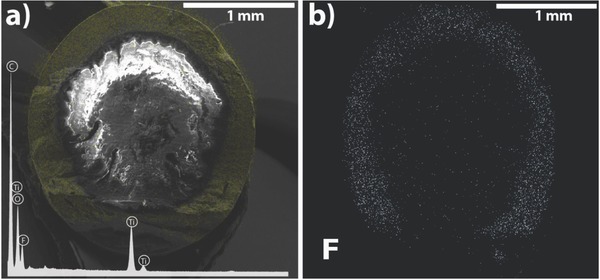
SEM‐EDX mapping image showing the presence and distribution of Ti (yellow) in the PMMA‐POF coating and inset of the EDX spectrum with the identification of the detected elements (a); mapping EDX of fluorine (F) distribution over the TiO_2_/PVDF coating (b).

The energy dispersive X‐ray spectroscopy (EDX) spectrum and mapping images obtained from a cross‐section of the 50 w/w% TiO_2_/PVDF coated POF (two dips) are shown in **Figure** **3**.

The obtained coating has a constant thickness around the PMMA core (Figure 3), which is in good agreement with the images obtained by optical microscopy. Additionally, Figure 3a shows a good interface between the coating and the optical fiber (indicative of a good adhesion) as well as the homogeneous dispersion of the TiO_2_ nanoparticles over the whole coating layer.

In the EDX spectra performed to the TiO_2_/PVDF coating (Figure S2, Supporting Information), carbon (C, the concentration of 19.6 ± 1.6) and fluorine (F, the concentration of 35.4% ± 1.5%) were identified. These elements are related to PVDF. Further, the presence of the TiO_2_ nanoparticles is confirmed by the titanium (Ti) and oxygen (O) peaks, corresponding to a TiO_2_ concentration of 45% ± 3% (obtained from the sum of  Ti and O). Also, by EDX mapping it was possible to observe a good dispersion of Ti (represented in yellow) without the evidence of nanoparticles cluster or agglomeration. The TiO_2_/PVDF weight ratio (45/55%) estimated by the EDX is in good agreement with the experimental amount of TiO_2_ and PVDF (50/50%). Regarding Figure 3b, the white dots represent an SEM‐EDX mapping of F, which also indicates the homogeneous and uniform distribution of the fluorinated coating (PVDF) around all the PMMA core.

### 2.2 Light Output Measurements

**Figure 4:**
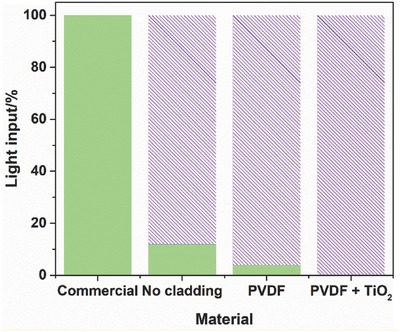
Artificial sunlight input (green) and loss of light (magenta pattern).

A detector measured the light transmission in a single POF, as a function of the different coatings at the 6 cm end tip (**Figure** **4**).

The measurements performed at the end tip of the fiber with different coatings allow estimating the reflected light propagated along the 60 cm of fiber until its end tip and the refracted/absorbed light. Before the cladding removal, the incident light (9.8 mW cm^−2^) suffered total reflection inside the fiber, and it was transmitted to the end tip, where the detector was placed. For the sample without cladding, only 12% of the incident light reached the end tip of the fiber, as 88% of the total light input was refracted by the naked core surface. When the clad‐free fiber was coated with PVDF, the transmitted light at the end of the tip decreased to 4%, which means that 8% of the light was absorbed by the PVDF coating, compared to the sample without cladding, as also confirmed by other works.^21^, ^68^ The incorporation of TiO_2_ nanoparticles exhibited a total absorption of light, with no transmission of the light to the tip of the fiber. This complete absorption of the light by the photocatalytic coating was also reported by Peill and Hoffmann.^77^ The light is propagated through the optical fiber due to total internal reflection because the core material (PMMA) possess a higher refractive index than the fluorinated polymer of the cladding. Since PMMA and PVDF exhibit a similar refractive index, 1.5 and 1.4, respectively,^78^, ^79^ the light propagated along the fiber is refracted when in contact with the PVDF coating. Also, as the refractive index of TiO_2_ (2.4–2.8) is higher than that of the PMMA (1.5), it is likely that the total internal reflection would not take place when the PMMA optical fiber is coated with TiO_2_.^79^ Therefore, the TiO_2_/PVDF coating receives all the input visible radiation (totally refracted), which matches one of the most important characteristics of a photocatalytic system.

### 2.3 Photocatalytic Results

**Figure 5:**
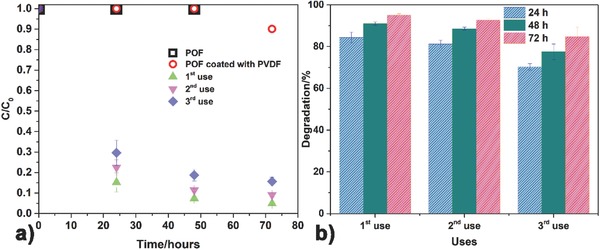
Photocatalytic degradation, *C*/*C*_0_ versus time (a) and degradation efficiency versus number of uses (b), of 5 mg L^−1^ of CIP for 72 h under artificial sunlight using the 50 w/w% TiO_2_/PVDF‐coated polymeric optical fibers.

The photocatalytic efficiency of the 50 w/w% TiO_2_/PVDF‐coated (sample with one dip) POF was assessed in the degradation of a solution of CIP (5 mg L^−1^) under artificial sunlight. The absorption peak of CIP at 275 nm was monitored as a function of time (72 h) with a UV–vis spectrophotometer. Additionally, the noncoated POF and the POF coated with PVDF (without TiO_2_) were also tested as controls (**Figure** **5**a).

The results obtained with the controls (absence of nanocatalyst) show that the CIP solution is photostable under artificial sunlight as no photolysis occurred. Additionally, CIP was not adsorbed by the uncoated POF, and only 10% was adsorbed by the PVDF coated POF. These results show that the decrease of the CIP in solution is attributed to its degradation. Regarding the TiO_2_/PVDF coated POFs, after 72 h of irradiation 95% of CIP in solution was degraded in the first use. In the third use and for the same irradiation time, 84% of CIP was degraded (corresponding to an efficiency loss of ≈11%), likewise the apparent reaction rate also decreased from 0.04 h^−1^ in the first use to 0.02 h^−1^ in the third use (**Table** **1**).

**Table 1:** Degradation of CIP, calculated final concentration, and the apparent reaction rate constant (*k*) after 72 h of artificial sunlight

Uses	Degradation		
	Degradation [%]	Final concentration [mg L^−1^]	*k* [h^−1^]
1st use	95.1 ± 0.7	0.2	0.04
2nd use	91.0 ± 0.8	0.5	0.03
3rd use	84.3 ± 2.6	0.8	0.02

EDX spectra and mapping images were also performed to the surface of the TiO_2_/PVDF/POF (Figure S3, Supporting Information) to understand the efficiency loss. In Figure S3 in the Supporting Information, it is possible to identify Ti (from TiO_2_ nanoparticles) homogeneously present over all the coating surface. The TiO_2_ plays a double critical role. On the one hand, it promotes the photocatalytic, on the other hand, TiO_2_ enhances the hydrophilicity^80^ of the coating which promotes higher interaction between the pollutant and the photocatalytic nanoparticles, favoring the adsorption of CIP. In this context, the detachment of the poorly attached nanoparticles to the coating surface often contributes to a decrease in the photocatalytic efficiency, in good agreement with previous works.^74^, ^75^, ^76^ Additionally, other works have reported the enhanced hydrophilicity of TiO_2_/PVDF nanocomposites when compared with the pristine polymer.^81^, ^82^ The presence of TiO_2_ nanoparticles increases the hydrophilicity of the nanocomposites,^83^, ^84^ especially after light irradiation.^85^, ^86^ The changes in the wettability of the composites are related both to the topological and chemical structure of the surface.^82^ Likewise, in the present work, the enhanced hydrophilicity is also caused by the increased roughness of the surface triggered by the TiO_2_ nanoparticles and by the presence of hydrophilic functional groups like oxygen originated on the nanoparticles surface upon light irradiation.^87^

### 2.4 Comparison with Related Systems

Until recently, not many researches were devoted to optical fibers applied in photocatalysis and any report using polymeric coated POF or degrading CIP was found. Thus, the present results cannot be compared with the literature. Related works have reported on a solar‐powered photocatalytic reactor (4.2 L),^77^ in the degradation of 4‐chlorophenol using 573 quartz optical fibers (QOFs) with 1 mm of diameter, and 1.30 m long coated with 15 wt% TiO_2_ (30 cm) under sunlight. The experiment was performed for 2 d with a sunlight exposure of 7 h d^−1^. However, the number and the length of TiO_2_ coating over the QOF are much higher when compared with our work (only 20 fibers with a 6 cm TiO_2_/PVDF coating). Additionally, this study did not provide any evaluation of the reusability of the TiO_2_/QOF. Reusability assays were performed with 30 pieces of side‐glow QOF,^22^ using a 5% Fe‐TiO_2_/polyethylenimine coating in the degradation of rhodamine B. The durability of the coating was tested under UV with a decrease of efficiency of 82%–49% after 42 h of operation. The inefficient attachment of the nanoparticles probably causes this 33% of efficiency loss, in good agreement with our SEM results previously discussed (Figure 1).

Regarding the application of polymeric optical fibers in photocatalysis, 15 POFs with 1 mm of diameter were coated with a 5% TiO_2_ (40 cm) aqueous suspension^6^ and tested under visible light in the photocatalytic degradation of trichloroethylene (reactor of 650 mL). The studied compound was completely degraded after ≈40 min under an intensity of 32 mW cm^−2^ and 12 cm of photocatalytic coating. However, the authors did not assess the reusability of the material.

In the present study, a degradation of 95% of CIP was reached within 72 h under artificial sunlight using 20 POF with TiO_2_ nanoparticles immobilized in PVDF. However, it is important to highlight that the immobilization of the nanoparticles into a substrate implies the loss of surface area and the reduction of active adsorption sites as well as mass transfer limitations.

**Figure 6:**
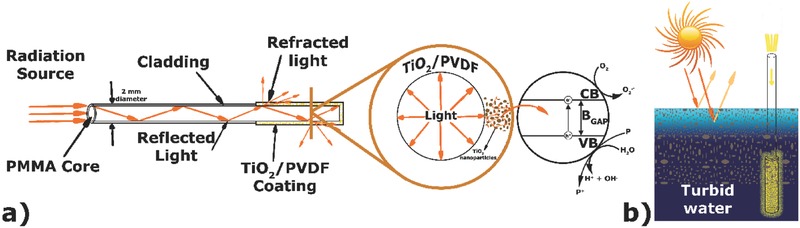
Conceptual representation of the PMMA optical fibers coated with TiO_2_/PVDF and the different optical phenomena occurring during irradiation in photocatalytic applications (a). Schematic representation of the application of POF in the photocatalytic treatment of turbid water (b).

In the presented system (**Figure** **6**), the radiation is transmitted throughout the PMMA core by reflection, and the refracted light (in the absence of the cladding) reaches the TiO_2_ nanoparticles immobilized into the PVDF coating. In this way, regardless the turbidity or the remoteness of the polluted water, the photocatalytic process can take place because the TiO_2_/PVDF coating is not irradiated from the outside (where the medium can be a limiting factor). In this case, the radiation is provided by the inner core of the POF that is in direct contact with the produced photocatalytic coating. Additionally, the radiation can be transported to deep water using bundles of TiO_2_/PVDF coated POF. The system herein presented avoids the hindrances that a turbid medium may impose to the photocatalytic process and allows the degradation of pollutants in places where radiation does not penetrate, such as in ground and deep water.

## 3 Conclusion

In this work, a TiO_2_/PVDF photocatalytic coating was successfully immobilized on the surface of PMMA optical fibers by dip coating, and the hybrid composite was characterized. The optimization process showed that 15 w/w% of PVDF and a TiO_2_ concentration of 50 w/w% yielded the more uniform and robust coating. The coating thickness was also optimized obtaining a good homogeneity of the TiO_2_/PVDF coating around the PMMA core and proper distribution of the TiO_2_ along the coating and.

The total inputted radiation to the POF was delivered to the produced TiO_2_/PVDF coating, which is paramount to ensure the photocatalytic efficiency of the coating. The photocatalytic tests confirm the excellent performance of the newly developed system, showing degradation of 95% of CIP after 72 h of irradiation and a reutilization up to three times with an efficiency loss of ≈11%, which indicates that the proposed material has interesting reusability properties.

Thus, the present investigation confirms the suitability of the TiO_2_/PVDF coating for photocatalytic applications in water mediums with low or none radiation, opening a vast, new, and needed area of applicability for water remediation.

## 4 Experimental Section

*Coating of the Polymeric Optical Fibers*: First, according to the procedure used by Peill and Hoffmann,^88^ a coating solution consisting of an aqueous dispersion of 15% (w/w) TiO_2_ nanoparticles (P25—kindly provided by Evonik) was produced to assess the mechanical stability of the TiO_2_ nanoparticles coating on PMMA‐POF. According to the manufacturer, the TiO_2_ P25 nanoparticles have 21 nm diameter, a surface area of 50 ± 15 m^2^ g^−1^, and the anatase:rutile ratio is typically 70:30. The coating obtained with TiO_2_ nanoparticles was further compared to the polymeric coatings. For this, different polymeric coating solutions were prepared to evaluate the mechanical stability of the coatings. Therefore, various amounts of PVDF (1010/1001, Solef) were dissolved in 9.7; 9.5; 9, and 8.5 mL of *N*,*N*‐dimethylacetamide (DMA, Sigma‐Aldrich, purity ≥ 99.5%) to reach final concentrations of 3%, 5%, 10%, and 15% (w/w) of PVDF, respectively. Mechanical stirring was applied until complete polymer dissolution.

To optimize the photocatalytic performance of the PVDF‐based coatings, different amounts of TiO_2_ (25, 40, and 50 w/w%) were dispersed in DMA using an ultrasonic bath for 3 h. Afterward, the polymer was added to reach the final concentration of 15% (w/w) and the solution was mechanically stirred until complete polymer dissolution.

The end tip (6 cm) of the 2 mm PMMA optical fibers (Conrad) 60 cm long was roughened with a sandpaper no 80 (Buehler). Acetone was used to eradicate the cladding debris. The 6 cm end tip of the POF was dip coated with the selected coating solutions and withdrawn after 10 s. The dipped fibers dried at room temperature for 12 h. For different coating thicknesses, it was performed an increasing number (one, two, four, and six) of consecutive dipping, repeating the dipping procedure.

*Characterization of POF Coated with TiO2/PVDF*: The morphological characterization was assessed by SEM, using an FEI Quanta 650 field emission gun (FEG) microscope equipped with an INCA 350 spectrometer (Oxford Instruments) for energy dispersive X‐ray spectroscopy (EDX), for elemental and distribution analyses. To determine the coating thickness, microscopy (Optiphot‐100 50 W from Nikon; camera model EC3 from Leica) was used and each sample was analyzed 20 times (Image J—version 1.48). The adhesion of the coating to the optical fiber was assessed placing a tape strip (7 cm) longitudinally to the fiber coating and removed after 1 min, measuring the fiber weight before and after the test to estimate the coating mass loss. Optical microscopy was also used to evaluate the integrity of the coating. To assess the attenuation and to determine the amount of light absorbed by the coating, the difference between the input and the output of artificial sunlight was calculated. For that, the fibers (60 cm long) were stretched, one of the tips was placed next to the light source (9.8 mW cm^−2^), and at the opposite end, the light output was measured with a power detector (XLP12‐3S‐H2‐D0, from GENTEC Industries). These tests were performed in POF without cladding, with cladding, coating with PVDF, and with a 50% (w/w) TiO_2_/PVDF coating.

**Figure 7:**
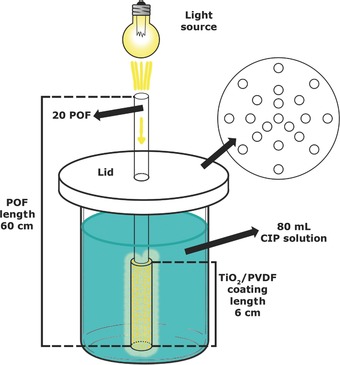
Schematic representation of the photocatalytic assays setup.

*Photocatalytic Degradation Experiments*: The photocatalytic assays were carried out using a sun simulator (AM1.5, Ingenieurbüro Mencke and Tegtmeyer GmbH) with a light intensity of 9.8 mW cm^−2^. A beaker with (80 mL) aqueous solution (5 mg L^−1^) of CIP, purchased to Sigma‐Aldrich, was used to test the photocatalytic activity of 20 POF with a 2 mm diameter POF bundle and 60 cm long—represented in **Figure** **7**.

The POFs dipped once in the 50 w/w% TiO_2_/PVDF solution were used for the photocatalytic assays. The light was focused at the top of the POF bundle and transmitted through the 60 cm of the fibers. The 6 cm of TiO_2_/PVDF coating was wholly immersed in the CIP solution. Between the CIP solution and the irradiation source, it was placed a polystyrene lid with through holes for the fibers to pass. The spacing between the fibers prevented exfoliation of the photocatalytic coating and the cover completely covered the CIP solution to avoid the irradiation to reach the solution except by the optical fibers. Before illumination, the solution with the immersed POF coated part was stirred in the dark for 30 min to achieve the adsorption–desorption equilibrium of the antibiotic onto the photocatalytic coating. Later, the POF were exposed to artificial sunlight for 72 h under constant magnetic stirring. Aliquots of 2 mL were withdrawn every 24 h. As controls, experiments were carried out in the absence of the photocatalytic coating. The photocatalytic degradation was determined by analyzing the decrease of the absorption peak of ciprofloxacin with a maximum at 278 nm by UV–vis spectrophotometry (Varian CARY‐100 UV–vis, Agilent). Three degradation cycles of 72 h each were performed to study the reusability of the photocatalytic coating. The CIP degradation fits a pseudo‐first order reaction, following the Langmuir–Hinshelwood model, represented by Equation (1).^8^

(1) $$\ln\frac{C}{C_{0}}\;=\;-kt$$

where *C*_0_ and *C* represent the concentration of the pollutants at time 0 and at time *t*, respectively, and *k* is the first‐order constant of the reaction. The degradation rate was calculated from the slope of the exponential curve of the concentration plot as a function of time.

## Conflict of Interest

The authors declare no conflict of interest.

## Supporting information

Supplementary
