# Supplementary material for: Reusable Photocatalytic Optical Fibers for Underground, Deep‐Sea, and Turbid Water Remediation
Source: Glob Chall. 2018 Feb 21;2(3):1700124. doi: 10.1002/gch2.201700124 (PMC6607349; doi:10.1002/gch2.201700124)
Supplement: Supplementary file 1 — Supplementary [file GCH2-2-1700124-s001.pdf]

Copyright WILEY-VCH Verlag GmbH & Co. KGaA, 69469 Weinheim, Germany, 2018.

Global Challenges
Open Access

Supporting Information

for *Global Challenges*, DOI: 10.1002/gch2.201700124

Reusable Photocatalytic Optical Fibers for Underground, Deep-Sea, and Turbid Water Remediation

Sara Teixeira, Bruno Magalhães, Pedro M. Martins, Klaus Kühn, Lluís Soler, Senentxu Lanceros-Méndez,* and Gianaurelio Cuniberti*

Supporting Information

Reusable photocatalytic optical fibers for underground, deep-sea and turbid water remediation

Sara Teixeira, Bruno Magalhães, Pedro Martins, Klaus Kühn,a Lluís Soler, Senentxu Lanceros-Méndez,* Gianaurelio Cuniberti*

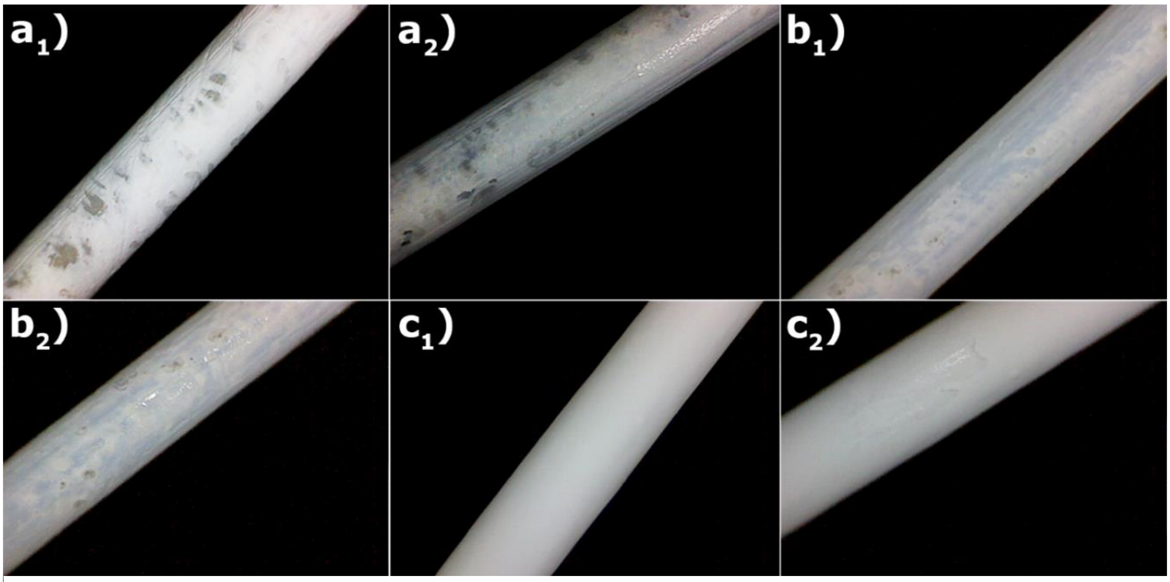

Figure S1. Tape test of the coating containing 15 w/w % of PVDF and 25 w/w % of TiO_2 before a_1) and after the tape test a_2); 40 w/w % of TiO_2 before b_1) and after the tape test b_2) and 50 w/w % of TiO_2 before c_1) and after the tape test c_2).

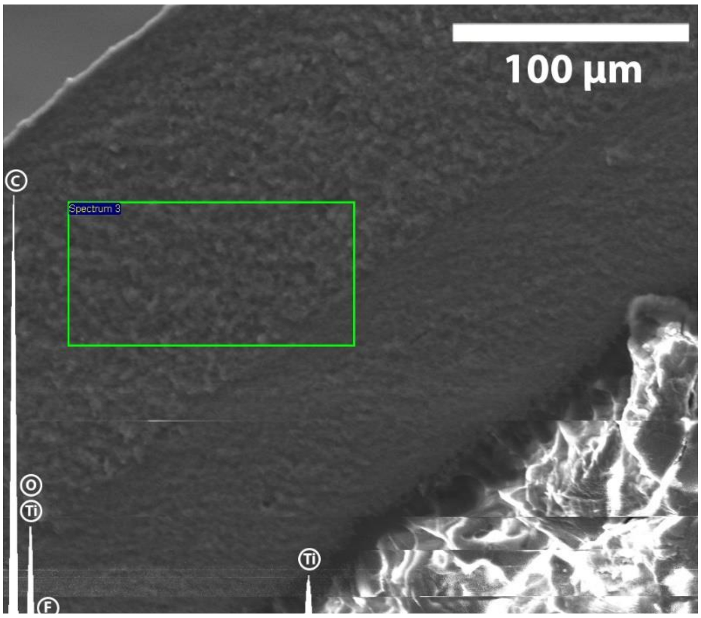

Figure S2. SEM-EDX of the TiO_2/PVDF coating. The elemental analysis corresponds to the elements identified in the green box.

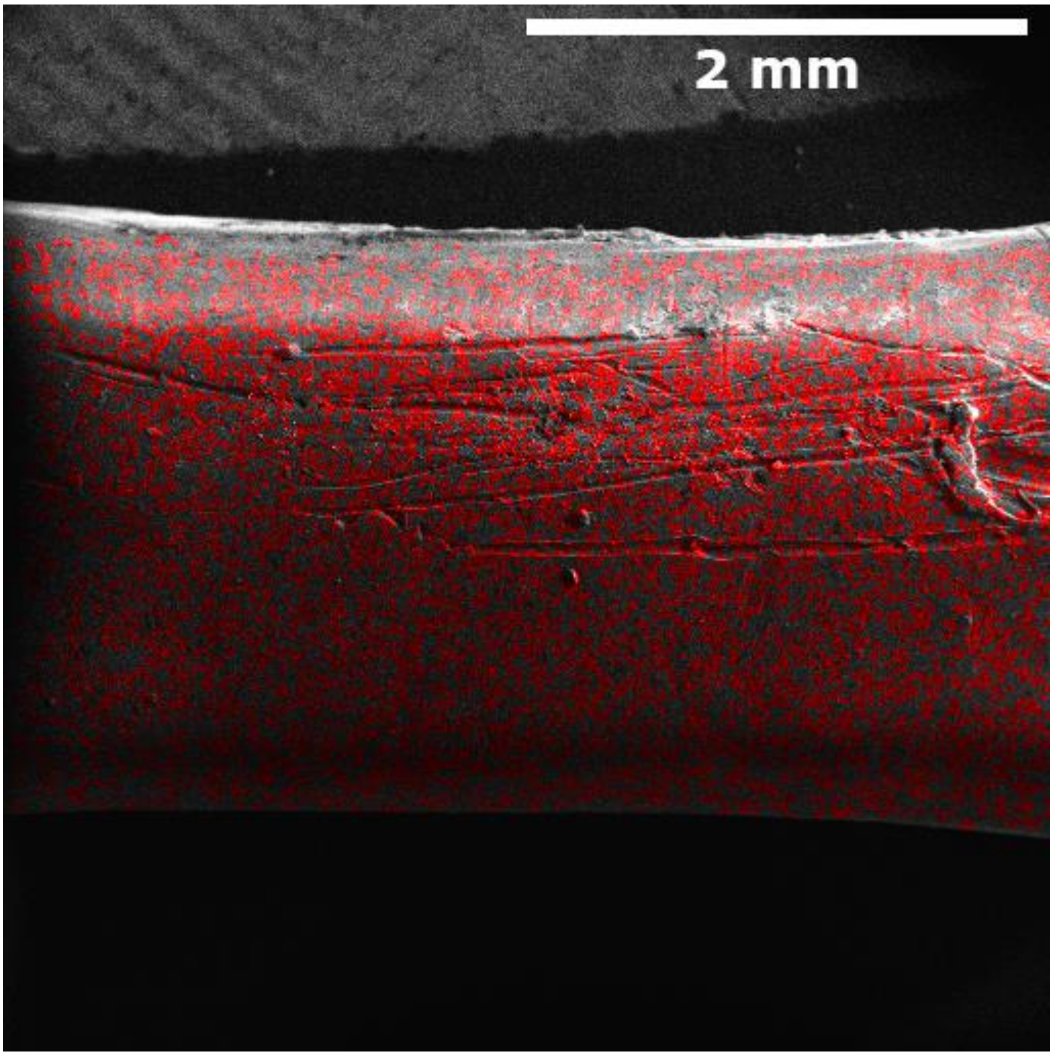

Figure S3. Surface SEM-EDX mapping of the 50 w/w % TiO_2/PVDF POF. Titanium (Ti) identified in red.
